# Reactive Nitrogen–Dominant Plasma Accelerates Diabetic Wound Healing Through Regulated Angiogenesis and TGF‐β Normalisation

**DOI:** 10.1111/iwj.70949

**Published:** 2026-06-02

**Authors:** Yu‐Pin Cheng, Tien‐Yang Chou‐Huang, Chih‐Chia Lee, Hua‐Lin Chen, Kuang‐Yao Cheng, Mu‐Chien Wu, Shih‐Sen Huang, Jiun‐Wen Guo, Jong‐Shinn Wu

**Affiliations:** ^1^ Department of Mechanical Engineering National Yang‐Ming Chiao Tung University Hsinchu Taiwan; ^2^ Department of Biological Science and Technology National Chiao Tung University Hsinchu Taiwan; ^3^ Department of Dermatology Cathay General Hospital Taipei Taiwan; ^4^ National Center for Instrumentation Research National Institutes of Applied Research Hsinchu Taiwan; ^5^ Department of Medical Research Cathay General Hospital Taipei Taiwan

**Keywords:** angiogenesis, diabetic foot, plasma gases, transforming growth factor beta, wound healing

## Abstract

Chronic diabetic wounds persist because of impaired angiogenesis, dysregulated transforming growth factor beta activity and delayed matrix remodelling. Non‐thermal atmospheric pressure plasma therapy represents a potential non‐pharmacologic approach to overcome these barriers. This study compared reactive nitrogen–dominant and reactive oxygen–dominant plasma exposures under identical apparatus conditions in a diabetic wound model. A universal plasma jet operated with nitrogen or argon gas was applied to streptozotocin‐induced diabetic rats. Wound area reduction and time to 90% closure were quantified. Histological evaluation assessed re‐epithelialisation and collagen deposition and immunohistochemistry measured angiogenesis using cluster of differentiation 31 staining and transforming growth factor beta expression. Nitrogen plasma treatment demonstrated sustained improvement in wound reduction relative to diabetic controls and reached 90% closure on day 19, whereas argon plasma reached this threshold on day 24 and diabetic controls exceeded 30 days. Nitrogen plasma was also associated with an earlier, self‐limited angiogenic response characterised by an early cluster of differentiation 31 peak on day 6, together with patterns consistent with enhanced collagen maturation and earlier normalisation of transforming growth factor beta expression. Overall, these findings suggest mechanistic differences between reactive nitrogen–dominant and reactive oxygen–dominant plasma exposures in regulating angiogenesis and matrix remodelling during diabetic wound repair. These results indicate that plasma gas chemistry may influence wound‐healing trajectories, supporting the potential of plasma therapy as a translational adjunct approach for difficult‐to‐heal wounds.

## Highlights

- Nitrogen‐ and argon‐based plasmas improved diabetic wound repair.
- Plasma gas chemistry influenced wound‐healing trajectories and repair patterns.
- Nitrogen‐dominant plasma was associated with physiologic angiogenic timing and earlier TGF‐β normalization.
- Argon‐dominant plasma showed wound‐healing patterns consistent with oxidative pathway–mediated responses.
- Cold plasma may serve as a non‐pharmacologic adjunct for difficult‐to‐heal wounds.

## 1 Introduction

Diabetes mellitus (DM) is a chronic metabolic disorder characterised by sustained hyperglycemia, resulting from either insulin deficiency (Type 1 DM) or insulin resistance (Type 2 DM) [1, 2]. The global prevalence of DM continues to rise, posing significant clinical and economic burdens on healthcare systems [3, 4, 5]. Impaired wound healing of the lower extremities represents one of the most devastating complications of DM, often leading to diabetic foot ulcers (DFUs), infection and eventual limb amputation and remains a major unmet clinical challenge [6, 7, 8]. Beyond standard debridement, offloading and dressings, there remains a paucity of non‐pharmacologic, clinic‐deployable physical adjuncts that can reliably accelerate closure while minimising infection and scarring risks.

Delayed healing of diabetic wounds is attributed to a multifactorial pathogenesis involving vascular dysfunction, neuropathy and chronic inflammation [7]. Hyperglycemia further impairs wound repair by prolonging the inflammatory phase and promoting oxidative stress through excessive reactive oxygen species generation and reduced antioxidant defence capacity, thereby disrupting cellular repair processes and contributing to chronic non‐healing wounds [9, 10]. Macroangiopathy and microangiopathy both contribute to compromised perfusion and oxygenation at the wound site, thereby sustaining inflammation and impairing re‐epithelialisation [7, 11]. Furthermore, nitric oxide (NO)—a key signalling molecule involved in vasodilation and angiogenesis—is diminished in diabetic tissues, contributing to delayed wound closure [12, 13]. Beyond its vasodilatory role, NO also functions as a multifunctional signalling mediator that regulates inflammatory responses, keratinocyte proliferation, microcirculatory dynamics and other cellular processes essential for skin homeostasis and wound repair [14, 15, 16]. Restoring NO bioavailability has been shown to enhance diabetic wound healing by promoting endothelial cell proliferation, neovascularisation and collagen synthesis [17, 18, 19]. These observations position nitric‐oxide–relevant chemistry as a rational target for device‐based wound modulation.

Atmospheric pressure plasma jets (APPJs) have emerged as a novel, non‐thermal approach for enhancing wound healing due to their ability to generate biologically active species, modulate inflammation and stimulate tissue regeneration [20, 21]. Among various plasma sources, argon‐based APPJs have been the most widely investigated, primarily for their generation of reactive oxygen species (ROS) that promote antimicrobial activity, oxidative stress signalling and angiogenesis [20, 22, 23]. ROS such as hydroxyl radicals and superoxide anions act as secondary messengers in immune and non‐immune cells, driving oxidative bursts and pro‐healing responses in chronic wound environments [24, 25]. Importantly, gas chemistry is a modifiable exposure parameter that may tailor biologic responses at the wound bed.

In addition to ROS, reactive nitrogen species (RNS), particularly NO and its derivatives, play a critical role in wound healing processes. RNS have been implicated in angiogenesis, immune regulation and extracellular matrix remodelling [19, 26], suggesting that RNS‐dominant exposures could address NO deficits in diabetic wounds. Given the known deficiency of NO in diabetic wounds, nitrogen‐based APPJs—which produce substantial amounts of NO‐related species such as NO‐γ, NO‐β, the nitrogen second positive system and the nitrogen first negative system—offer a promising therapeutic alternative [27, 28, 29]. Despite the recognised benefits of APPJ‐based interventions, comparative in vivo studies evaluating the specific effects of nitrogen‐ versus argon‐based plasmas in diabetic wound healing remain scarce. To our knowledge, this is the first systematic in vivo comparison of nitrogen‐ versus argon‐dominant plasmas in a diabetic wound model under identical apparatus conditions, addressing a critical gap in plasma medicine research.

In this study, we employed a universal atmospheric pressure plasma jet that can operate with either nitrogen or argon gas under identical electrical and flow parameters to directly compare reactive nitrogen–dominant and reactive oxygen–dominant exposures in streptozotocin‐induced diabetic rats. We postulated that gas chemistry would determine distinct wound‐healing trajectories by influencing angiogenesis and transforming growth factor beta regulation, rather than producing a simple dose‐dependent effect. This investigation was designed to provide preclinical evidence and mechanistic insight supporting plasma therapy as a safe, non‐pharmacologic and clinically translatable approach for diabetic wound management.

## 2 Materials and Methods

### 2.1 Study Design, Ethics and Reporting

This preclinical study compared nitrogen‐ and argon‐based APPJs for diabetic wound healing in rats using a two‐phase design with prespecified histology/IHC timepoints. All procedures complied with institutional and national regulations (IACUC Approval No. NCTU‐IACUC‐106062). Animals were housed under controlled conditions with free access to food and water. Randomisation was used for group allocation and wound/histology/IHC quantification was performed by blinded investigators. Reporting follows key ARRIVE 2.0 items.

### 2.2 Plasma Jet Apparatus and Operating Conditions

A custom universal APPJ platform with interchangeable argon or nitrogen gases was used. The device comprised a quartz dielectric tube with a slot‐shaped stainless‐steel grounded electrode and an external aluminium‐tape powered electrode in a DBD‐like configuration. The power supply delivered a 20 kHz, 15 kVpp sinusoidal waveform. Ultra‐high‐purity gases (99.999%) were supplied at 30 slm; nozzle‐to‐tissue distance was fixed at 5 mm. Key operating parameters are summarised in Table 1.

**TABLE 1:** Key operating parameters of the universal APPJ system.

Parameter	Value
Working gas	Argon^a^ or Nitrogen^a^
Frequency (kHz)	20
Voltage (kV_pp_)	15
Waveform	Sinusoidal
Flow rate (slm)	30
Humidity (%)	50–70

^a^ Impurity: O_2_ 2.0 ppm, H_2_O 0.91 ppm.

### 2.3 Plasma Diagnostics and Safety Monitoring

Voltage and current were monitored with a high‐voltage probe and Rogowski coil; average power was derived from charge–voltage Lissajous figures (100 cycles). Optical emission spectroscopy (0.3 nm resolution) characterised plasma species. Gas temperature 5 mm downstream remained < 41°C.

### 2.4 Diabetic Rat Model

Type 1 diabetes was induced in male Sprague–Dawley rats (8 weeks, 275–300 g) by streptozotocin (60 mg/kg, i.p.) after fasting. Animals with glucose > 300 mg/dL 1 week later were deemed diabetic; insulin was given if glucose exceeded 500 mg/dL to prevent fatal weight loss.

### 2.5 Wound Creation and Treatment Protocol

Under isoflurane anaesthesia, a full‐thickness 21‐mm circular dorsal wound was created using a sterile biopsy punch (Day 0). Plasma treatment was initiated immediately after wound creation and applied for 150 s per day with a nozzle standoff distance of 5 mm. During treatment, the plasma jet was scanned concentrically across the wound surface and the scanning motion was performed manually by the operator following a standardised circular pattern to ensure uniform plasma exposure. Animals received either nitrogen‐ or argon‐based APPJ treatment, while sham gas exposure and untreated wounds served as controls.

### 2.6 Experimental Groups and Timepoints

Phase I (pilot experiment) included four diabetic rats, each receiving three wounds assigned to one of the following conditions: Type 1 Diabetes, Nitrogen APPJ‐treated Type 1 Diabetes and Nitrogen gas‐treated Type 1 Diabetes.

In Phase II (main cohort), animals were randomly assigned to four experimental groups: G1 (Normal Self‐Healing), G2 (Type 1 Diabetes Self‐Healing), G3 (Type 1 Diabetes with Nitrogen Plasma Treatment) and G4 (Type 1 Diabetes with Argon Plasma Treatment) (*n* = 16 per group). Animals were sacrificed on Days 7, 14 and 21 (*n* = 4 per group per timepoint) for histological and immunohistochemical analyses. To evaluate early angiogenic responses, an additional 48 rats were included for short‐term timepoint analysis on Days 2, 3 and 6 (*n* = 4 per group per timepoint).

### 2.7 Wound Imaging and Closure Metrics

Daily digital images were analysed in ImageJ to compute wound area ratio (Day x/Day 0). Because late‐stage closure is hard to verify, time‐to‐90% closure was prespecified as a surrogate endpoint.

### 2.8 Tissue Processing, Histology and Immunohistochemistry

Wound tissues were fixed in 10% neutral‐buffered formalin, paraffin‐embedded and sectioned at 5 μm. H&E staining evaluated tissue architecture, re‐epithelialisation and inflammation; Masson's trichrome assessed collagen deposition. IHC employed anti‐CD31 for angiogenesis and anti‐TGF‐β1 for wound‐healing activity, with sections developed using the UltraView DAB system.

### 2.9 Image Analysis and Quantitative Endpoints

For each stained section, 10 random high‐power fields (HPFs, 400×) were analysed. Re‐epithelialisation (%) was calculated as (neo‐epidermal length ÷ wound‐edge distance) × 100. Inflammation was assessed as neutrophil + lymphocyte counts per HPF, angiogenesis as CD31‐positive microvessels per HPF and collagen fraction from Masson's images using ImageJ thresholds. Two blinded assessors quantified all fields; discrepancies > 10% were jointly reviewed.

### 2.10 Statistical Analysis

Data are presented as mean ± SD. Group differences were tested by one‐way ANOVA with multiplicity‐adjusted pairwise comparisons (Bonferroni/Holm). A two‐sided *p* < 0.05 was considered significant. For longitudinal wound‐area ratios, significance windows (days vs. diabetic control with *p* < 0.05) were summarised per treatment. Analyses were performed per animal using averaged HPFs per section.

## 3 Results

### 3.1 Plasma Characterisation

**FIGURE 1:**
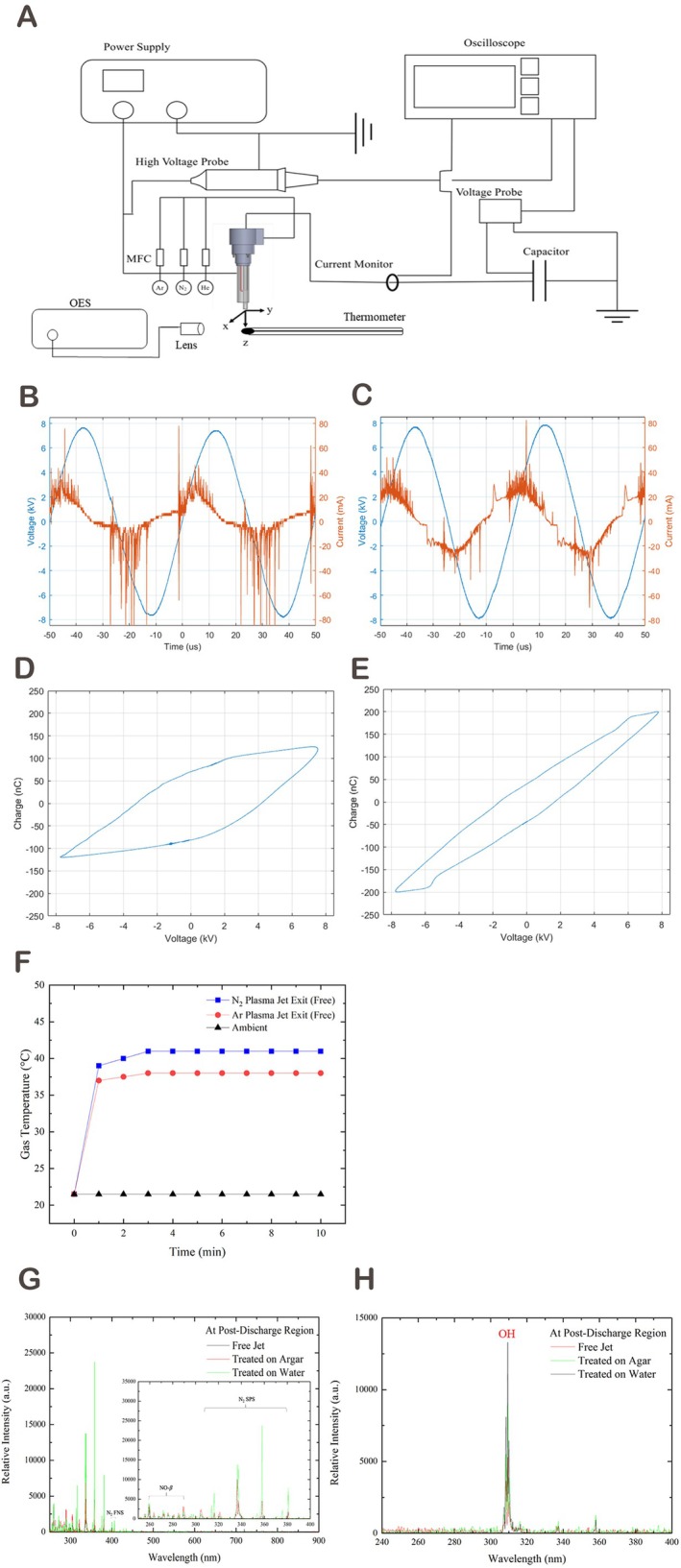
Physicochemical characterisation of APPJs. (A) Schematic diagram of the universal APPJ apparatus and electrical measurement instruments. The DBD‐like APPJ system consists of a quartz tube, a stainless‐steel grounded electrode (inside the quartz tube) and an annular aluminium tape‐powered electrode. The grounded electrode features a steel needle and slot‐shaped stainless steel, optimising gas flow distribution, increasing the contact area and enhancing the electric field strength at the discharge site. MFC, mass flow controller; OES, optical emission spectroscopy. (B) I‐V curve of nitrogen APPJ and (C) I‐V curve of argon APPJ, demonstrating the applied voltage and discharge current waveforms. (D) Lissajous figure of nitrogen APPJ (calculated average power: 30.19 W) and (E) Lissajous figure of argon APPJ (calculated average power: 19.81 W), averaged over 100 measurements. (F) Temperature profile of the universal APPJ measured over time at a distance of 5 mm from the jet exit, confirming its biocompatibility by maintaining a temperature of approximately 40°C. (G) OES analysis of nitrogen APPJ and (H) OES analysis of argon APPJ when applied to agar, water and in free jet conditions. Nitrogen FNS, nitrogen first negative system; Nitrogen SPS, nitrogen second positive system.

Figure 1A shows the schematic of the universal APPJ and measurement setup. The DBD‐like device comprised a quartz tube with a slot‐shaped grounded electrode and an annular powered electrode. Under identical operating voltage and gas flow conditions, the nitrogen APPJ generated a higher discharge current (~80 mA vs. ~50 mA) and greater average power (30.2 vs. 19.8 W) than the argon plasma. This difference reflects the intrinsic discharge characteristics of the two gases rather than intentional adjustment of plasma dosage, as the device was operated under identical electrical settings for both gases (Figure 1B–E), exhibited higher energy deposition under these operational conditions.

Gas temperatures 5 mm downstream remained < 41°C for both plasmas (Figure 1F), confirming non‐thermal safety. OES revealed distinct species profiles: nitrogen plasma showed strong NO‐γ/β and nitrogen bands, indicating abundant RNS, whereas argon spectra featured OH radicals, suggesting ROS dominance (Figure 1G,H). Together, these results demonstrate that nitrogen plasma produced higher discharge energy and RNS‐dominant emission signatures, whereas argon plasma exhibited lower discharge energy with ROS‐related emission features. Both plasmas operated within biocompatible temperature ranges.

### 3.2 Wound Healing Kinetics

**FIGURE 2:**
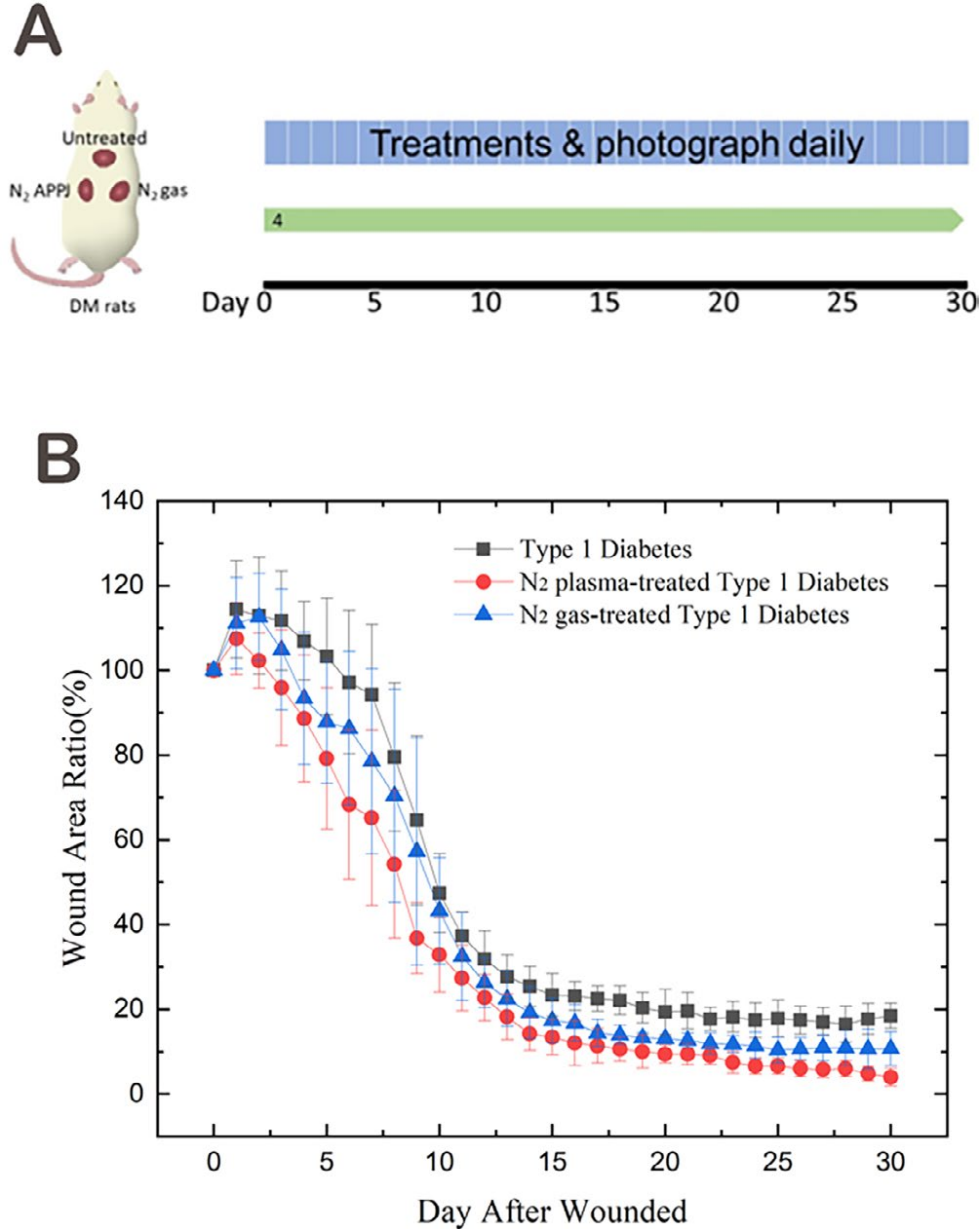
Animal experiment design and wound healing progression (A) Schematic representation of the experimental design. The first phase of the study included four diabetic rats, each receiving three full‐thickness wounds on the dorsum. The wounds were assigned to three treatment conditions: Type 1 Diabetes, Nitrogen APPJ‐treated Type 1 Diabetes and Nitrogen gas‐treated Type 1 Diabetes. Treatments were administered daily and photographs were taken throughout the 30‐day observation period. (B) The average wound area ratio over time for the three treatment conditions in four diabetic rats. Nitrogen APPJ‐treated wounds exhibited a faster reduction in wound area compared to nitrogen gas‐treated and untreated wounds, although the differences were not statistically significant. Data are presented as mean ± SD; *n* = 4 per group.

The initial evaluation of nitrogen APPJ efficacy was conducted by monitoring wound healing progression within the same diabetic rats. The therapeutic effects of nitrogen and argon APPJ treatments were assessed by evaluating wound contraction and tissue regeneration over time. Figure 2A depicts the experimental design, where diabetic rats received three full‐thickness wounds on the dorsum, each subjected to one of three conditions: nitrogen APPJ treatment, nitrogen gas exposure, or no treatment (control). This setup allowed for a direct comparison of the effects of nitrogen APPJ within the same animal. Figure 2B presents the wound healing kinetics, illustrating the temporal progression of wound closure under different conditions. The nitrogen APPJ‐treated wounds exhibited an accelerated reduction in wound area compared to both untreated wounds and those exposed only to nitrogen gas. However, the differences were not statistically significant.

**FIGURE 3:**
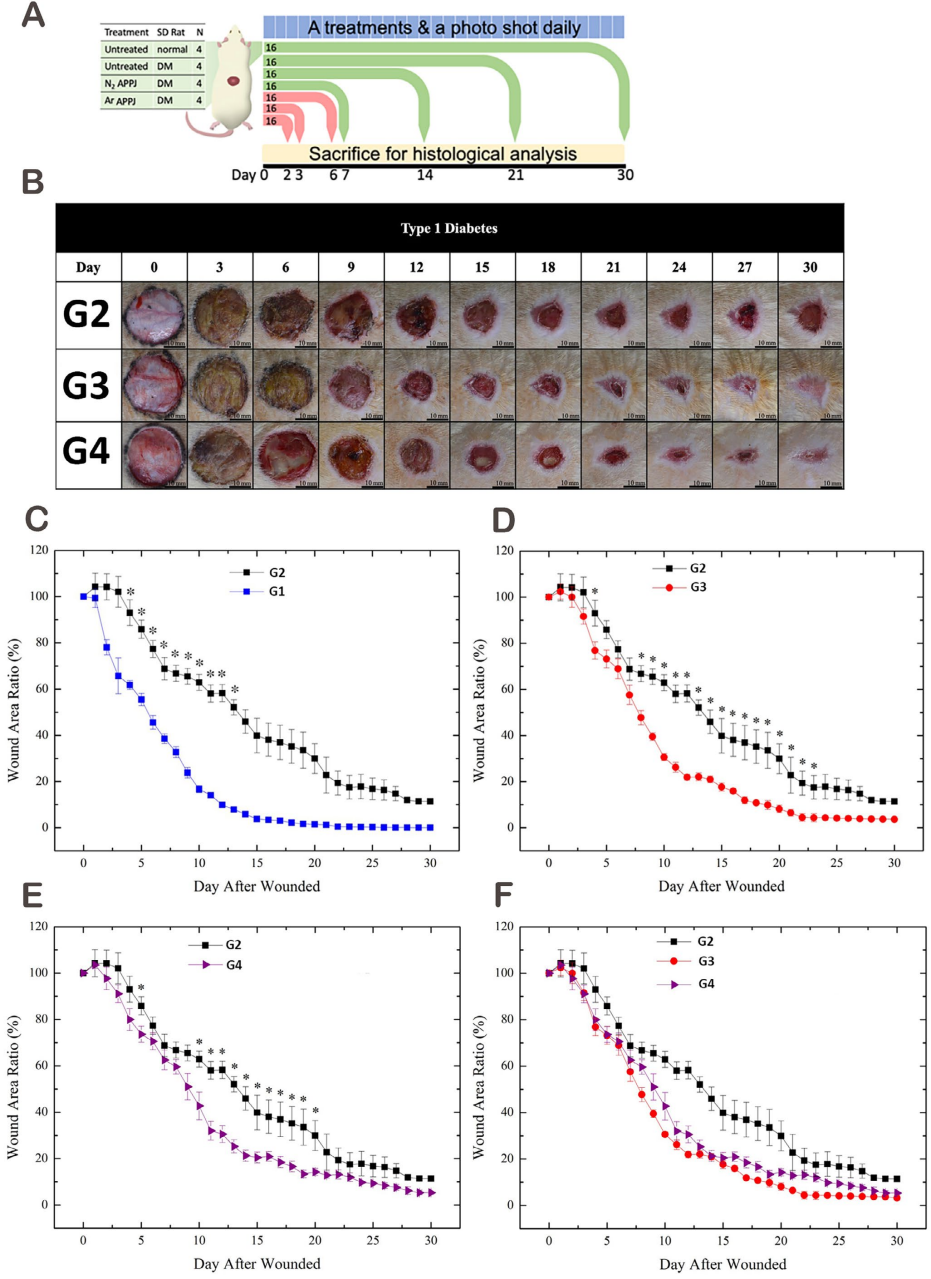
Wound healing kinetics in diabetic rats treated with APPJ. (A) Schematic representation of the experimental design. A total of 64 rats (four cohorts of 16 rats, divided into four groups) were included in the second phase of the study. An additional 48 rats (three cohorts of 16 rats across four groups) were used to assess angiogenic responses. (B) Representative wound images showing healing progression in nitrogen‐ and argon‐APPJ–treated groups compared with controls. Both APPJs accelerated closure relative to untreated diabetic wounds. (C–E) Wound contraction area ratios in normal, untreated diabetic, nitrogen APPJ–treated and argon APPJ–treated groups across time. (F) Comparative wound contraction in all groups. Both nitrogen‐ and argon‐APPJ significantly improved closure versus diabetic controls. Nitrogen‐APPJ exhibited a longer significance window and an earlier trend towards 90% closure; however, direct comparisons between nitrogen and argon were exploratory and not consistently significant. Data are presented as mean ± SD; *p < 0.05 indicates statistical significance. Group abbreviations used in the figures: G1, Normal Self‐Healing; G2, Type 1 Diabetes Self‐Healing; G3, Type 1 Diabetes with Nitrogen Plasma Treatment; G4, Type 1 Diabetes with Argon Plasma Treatment.

In the second phase, wound healing outcomes were compared between nitrogen‐ and argon‐APPJ treatments in a larger diabetic rat cohort (Figure 3A,B). Nitrogen APPJ reduced wound area by 30%–40% versus diabetic controls, most evident on days 8–12, while argon APPJ achieved a 20%–30% reduction on days 10–15. Both treatments enhanced re‐epithelialisation and produced drier wound surfaces compared with untreated wounds, with nitrogen APPJ showing a more consistent recovery trajectory.

Quantitative analysis confirmed delayed healing in untreated diabetic wounds, with stagnation in later stages (Figure 3C). Nitrogen APPJ accelerated contraction from days 4 and 8–23 (Figure 3D), whereas argon APPJ was significant from days 5 and 10–20 (Figure 3E). The significance window was longer with nitrogen (17 vs. 12 days). Time‐to‐90% closure was also shorter: day 19 for nitrogen, day 24 for argon and > 30 days for untreated wounds (Figure 3F).

Overall, both APPJs accelerated diabetic wound healing, but nitrogen exposure provided a more sustained effect and shortened closure by ~5 days, a clinically relevant benefit that may lower infection risk and reduce the burden of prolonged care.

### 3.3 Histological Evaluation

**FIGURE 4:**
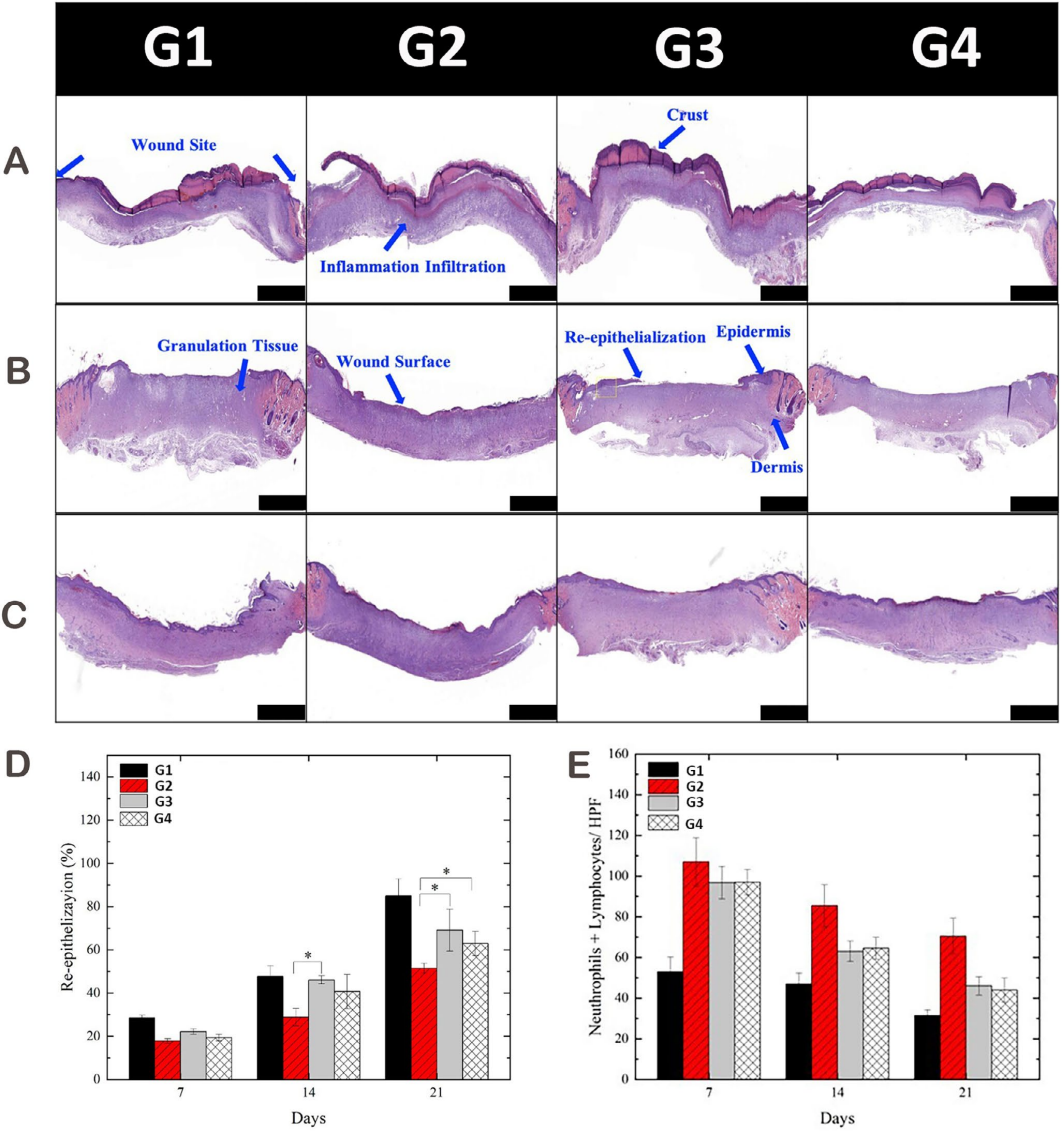
Histological evaluation of wound healing in normal and Type 1 diabetic rats with or without APPJ treatment. (A–C) Representative haematoxylin and eosin (H&E) staining of wound tissues at Day 7 (A), Day 14 (B) and Day 21 (C) across different experimental groups: Normal self‐healing, untreated Type 1 diabetic and Type 1 diabetic wounds treated with nitrogen or argon APPJ. Notable features include inflammation infiltration, crust formation, granulation tissue, re‐epithelialisation and dermal restoration. Scale bars: 2000 μm. (D) Quantification of re‐epithelialisation percentages over time. Nitrogen APPJ treatment significantly improved re‐epithelialisation compared to untreated diabetic wounds on Days 14 and 21 (*p < 0.05). (E) Quantification of inflammatory cell infiltration (neutrophils + lymphocytes per HPF) revealed reduced immune cell presence in plasma‐treated wounds over time. Data are presented as mean ± SD; *p < 0.05 indicates statistical significance. Group abbreviations used in the figures: G1, Normal Self‐Healing; G2, Type 1 Diabetes Self‐Healing; G3, Type 1 Diabetes with Nitrogen Plasma Treatment; G4, Type 1 Diabetes with Argon Plasma Treatment.

Histological examination using haematoxylin and eosin (H&E) staining was performed on Days 7, 14 and 21. In the early stage (Day 7), enhanced re‐epithelialisation and reduced inflammatory infiltration were observed in both APPJ‐treated groups compared to untreated diabetic wounds (Figure 4A). By Day 14, the nitrogen APPJ group displayed more advanced epithelial stratification and granulation tissue formation than other groups (Figure 4B). On Day 21, re‐epithelialisation in the nitrogen APPJ group nearly matched that of the healthy control, with statistically significant improvements over the untreated diabetic group (Figure 4C). Quantitative analysis confirmed that the nitrogen APPJ group consistently achieved higher re‐epithelialisation rates, followed by argon APPJ, with both outperforming the untreated group (Figure 4D). Inflammatory cell counts decreased over time in all groups, with APPJ‐treated wounds showing reduced neutrophil and lymphocyte infiltration compared to untreated wounds (Figure 4E). Histological analysis demonstrated that nitrogen‐APPJ showed higher re‐epithelialisation compared with diabetic controls and numerically exceeded argon‐APPJ; however, direct nitrogen versus argon differences were not consistently significant. By Day 21, the nitrogen‐APPJ group approached the healing profile of healthy controls, with restoration of epidermal continuity and reduced inflammation.

### 3.4 Collagen Deposition

**FIGURE 5:**
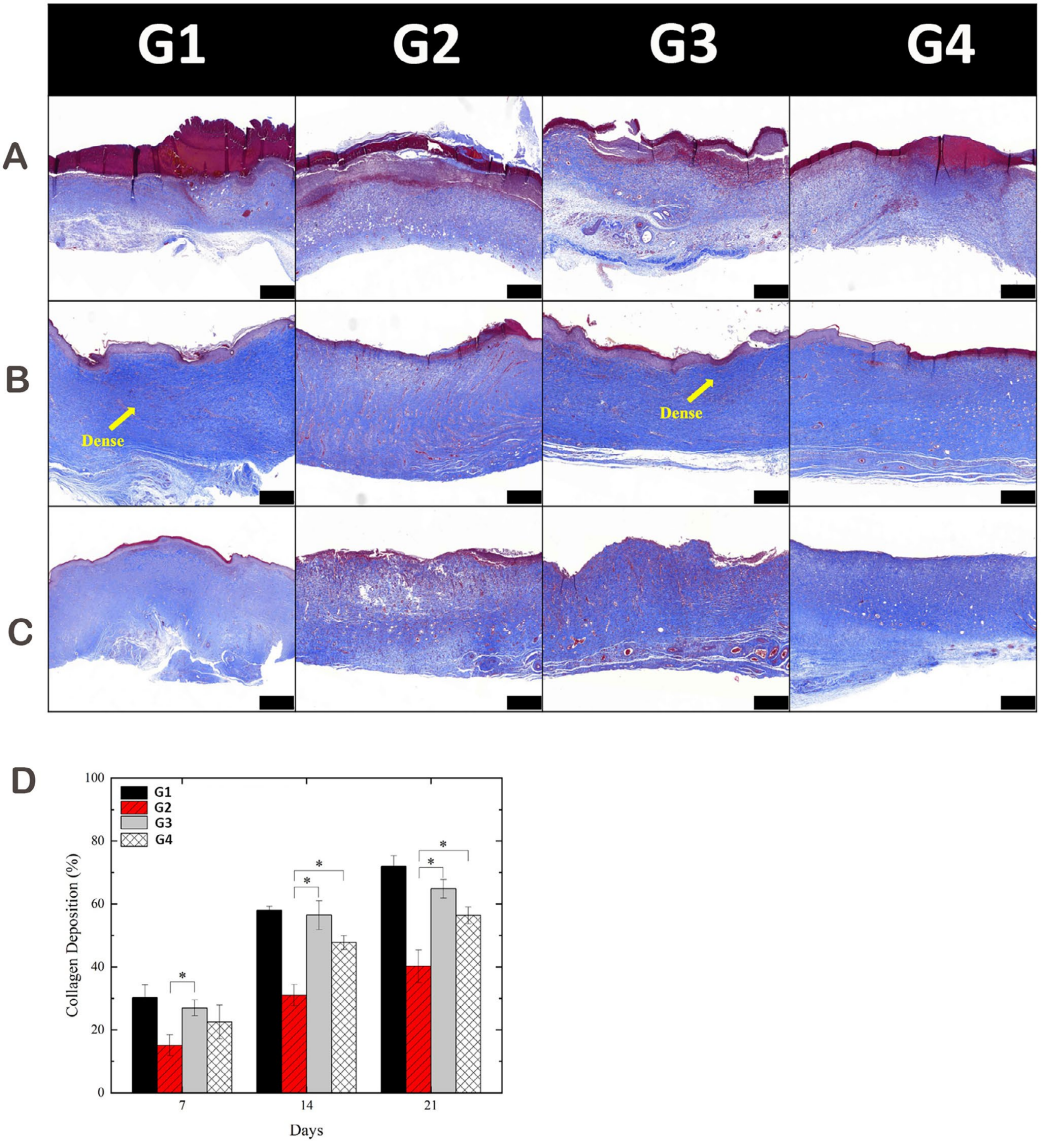
Collagen deposition analysis of wound tissues with and without APPJ treatment. (A–C) Masson's trichrome staining of wound sections at Day 7 (A), Day 14 (B) and Day 21 (C), showing collagen fibres in blue. Both APPJs promoted greater collagen deposition compared with untreated diabetic wounds. Scale bars: 500 μm. (D) Quantitative analysis confirmed that plasma exposure significantly increased collagen content versus diabetic controls. Nitrogen‐APPJ showed numerically higher levels than Ar‐APPJ across time points, but direct comparisons between the two APPJs were not consistently significant. Data are presented as mean ± SD; *p < 0.05 indicates statistical significance. Group abbreviations used in the figures: G1, Normal Self‐Healing; G2, Type 1 Diabetes Self‐Healing; G3, Type 1 Diabetes with Nitrogen Plasma Treatment; G4, Type 1 Diabetes with Argon Plasma Treatment.

Masson's trichrome staining was used to assess collagen deposition during wound healing. On Day 7, nitrogen‐APPJ–treated wounds showed significantly more blue‐stained collagen fibres than untreated diabetic wounds (Figure 5A). By Day 14, collagen maturation was enhanced in both APPJ‐treated groups, with nitrogen‐APPJ displaying a trend towards more organised collagen architecture (Figure 5B). On Day 21, wounds in the nitrogen‐APPJ group exhibited a mature collagen matrix approaching the profile of healthy controls (Figure 5C). Quantitative analysis confirmed that both APPJs increased collagen deposition compared with diabetic controls, with nitrogen‐APPJ showing numerically higher levels than argon‐APPJ across time points; however, direct nitrogen versus argon differences were not consistently significant (Figure 5D). Together, these findings suggest that APPJ exposure promotes earlier and more organised collagen maturation, thereby supporting dermal matrix remodelling in diabetic wound repair.

### 3.5 Angiogenesis Evaluation

**FIGURE 6:**
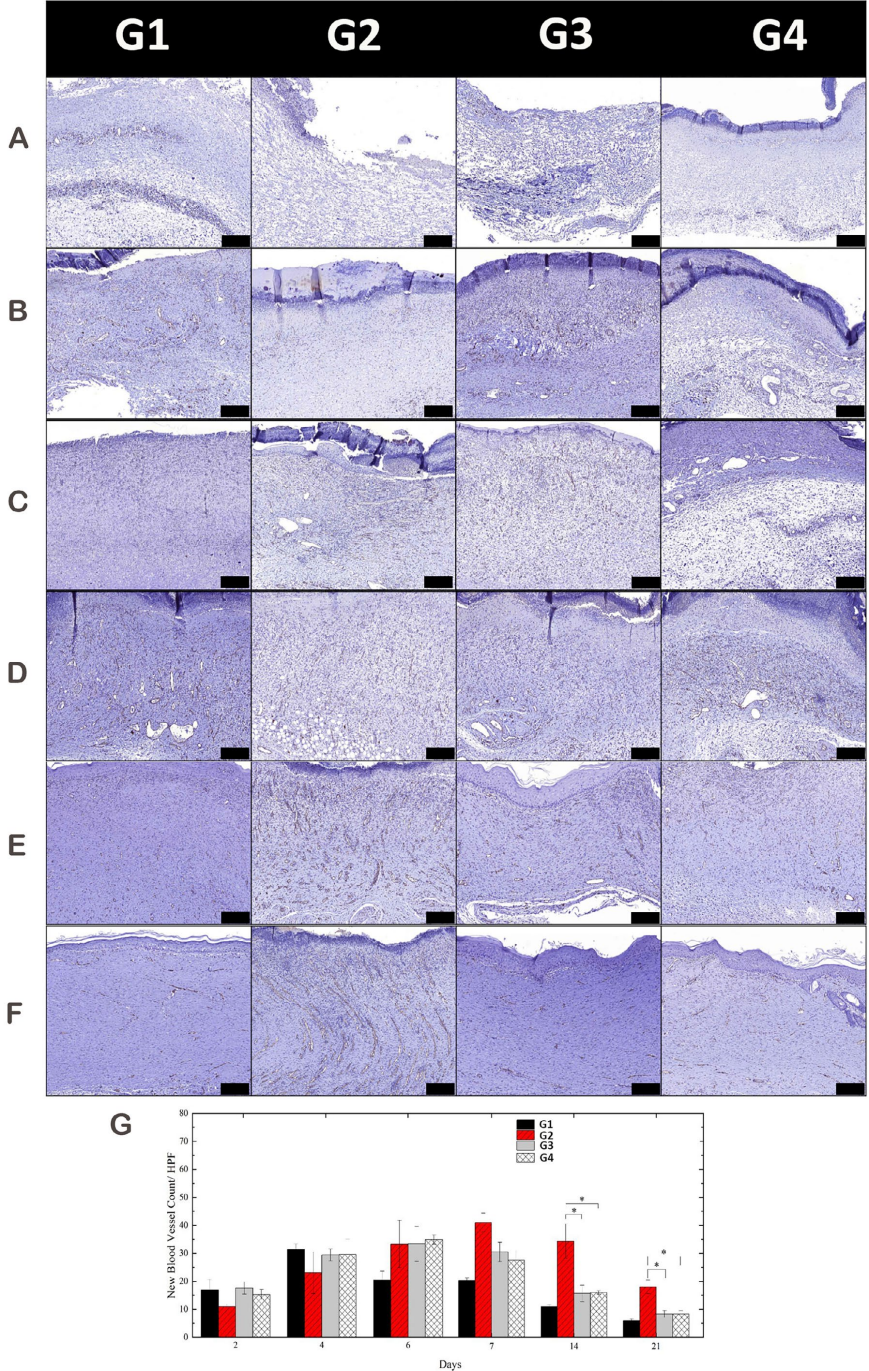
Immunohistochemical analysis of CD31 expression to assess neovascularisation during wound healing. (A–F) CD31 staining on Days 2 (A), 4 (B), 6 (C), 7 (D), 14 (E) and 21 (F) in normal, untreated diabetic and APPJ‐treated groups. CD31‐positive vessels peaked earlier in the nitrogen‐APPJ group (Day 6) and declined in a timely manner, while untreated diabetic wounds showed delayed and persistent angiogenesis (peak Day 7, persisting Day 21). Scale bars: 100 μm. (G) Quantitative analysis confirmed that both APPJs promoted earlier and better‐regulated angiogenesis compared with diabetic controls, with nitrogen‐APPJ more closely approximating the normal physiologic timeline. Data are presented as mean ± SD; *p < 0.05 indicates statistical significance. Group abbreviations used in the figures: G1, Normal Self‐Healing; G2, Type 1 Diabetes Self‐Healing; G3, Type 1 Diabetes with Nitrogen Plasma Treatment; G4, Type 1 Diabetes with Argon Plasma Treatment.

IHC staining for CD31, an endothelial cell marker, was conducted to assess neovascularisation. On Days 2, 4 and 6, a time‐dependent increase in CD31‐positive vessels was observed in all groups, with an earlier peak in the nitrogen‐APPJ group (Day 6) and a delayed peak in untreated diabetic rats (Day 7) (Figure 6A–C). By Days 14 and 21, vessel density declined in all groups; however, untreated diabetic wounds retained abnormally high CD31 expression, suggesting delayed angiogenic resolution (Figure 6E,F). Quantification indicated that nitrogen‐APPJ facilitated earlier and more regulated angiogenesis, more closely approximating the normal physiological timeline (Figure 6G). This alignment with physiologic angiogenic kinetics—earlier initiation with timely resolution—contrasted with the prolonged angiogenesis observed in diabetic controls. Timely and regulated angiogenesis is critical in preventing chronic ulcer persistence, underscoring the translational relevance of modulating gas chemistry in APPJ‐based therapy.

### 3.6 Wound Healing Activity and Potential Scarring

**FIGURE 7:**
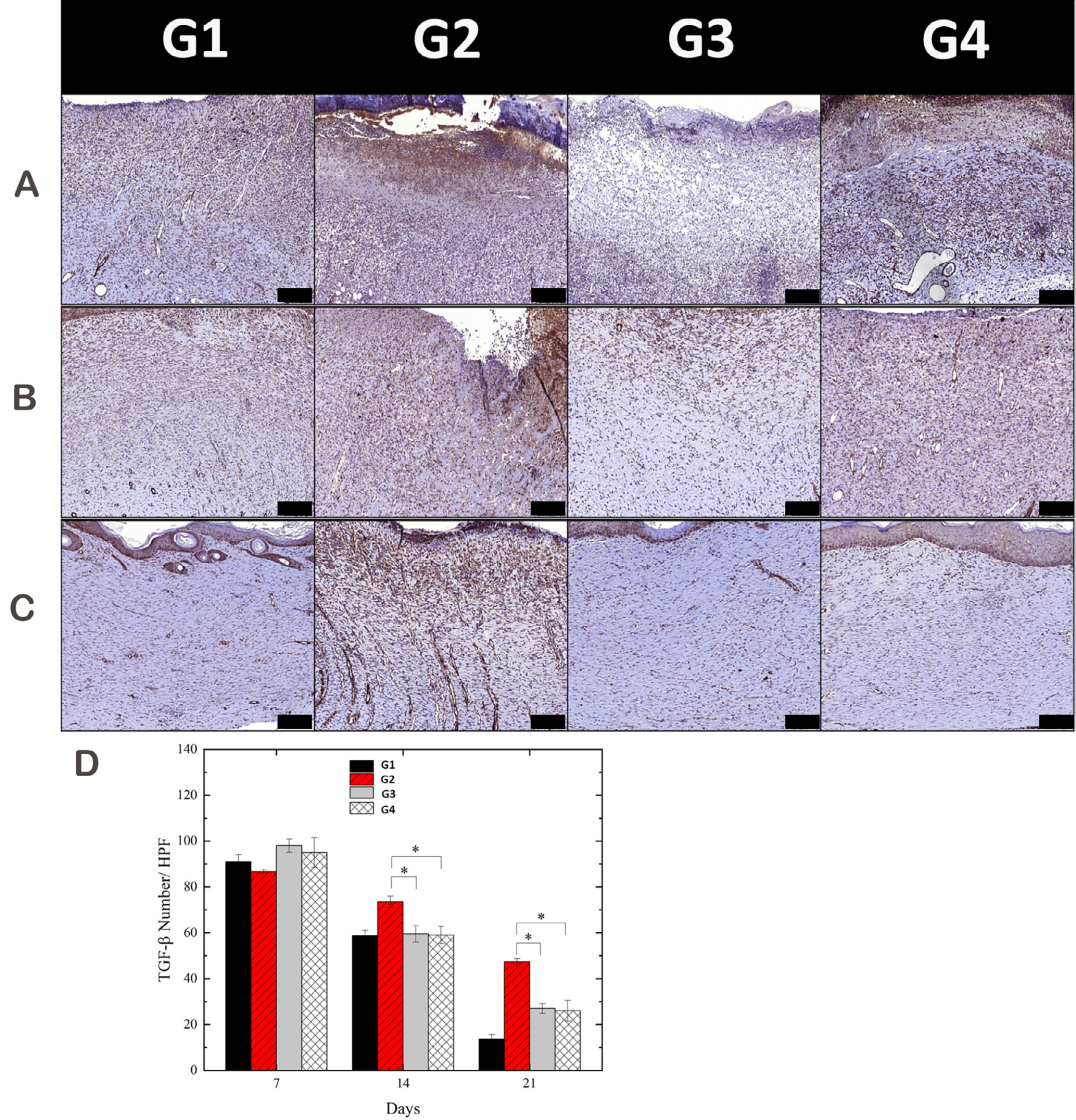
Immunohistochemical analysis of TGF‐β expression in wound tissues to evaluate healing dynamics and fibrotic potential. (A–C) Immunohistochemical staining of TGF‐β at Day 7 (A), Day 14 (B) and Day 21 (C) in normal, untreated diabetic and APPJ‐treated diabetic groups. Plasma treatment facilitated earlier downregulation of TGF‐β compared with untreated diabetic wounds. Scale bars: 100 μm. (D) Quantitative analysis showed that APPJ exposure accelerated normalisation of TGF‐β expression relative to diabetic controls, with nitrogen‐APPJ showing a more pronounced trend towards earlier resolution. Timely normalisation of TGF‐β suggests a more regulated healing trajectory and a potentially lower risk of fibrosis or hypertrophic scarring. Data are presented as mean ± SD; *p < 0.05 indicates statistical significance. Group abbreviations used in the figures: G1, Normal Self‐Healing; G2, Type 1 Diabetes Self‐Healing; G3, Type 1 Diabetes with Nitrogen Plasma Treatment; G4, Type 1 Diabetes with Argon Plasma Treatment.

TGF‐β immunohistochemistry was performed to evaluate wound healing activity and potential scarring. On Day 7, elevated TGF‐β expression was observed in all healing wounds, with higher levels in the normal and APPJ‐treated diabetic groups (Figure 7A). By Day 14, expression had declined in both APPJ‐treated groups but remained elevated in untreated diabetic wounds (Figure 7B). On Day 21, TGF‐β levels were minimal in the APPJ‐treated groups but persisted in untreated diabetic wounds (Figure 7C). Quantitative analysis indicated that nitrogen‐APPJ facilitated earlier normalisation of TGF‐β expression relative to diabetic controls, whereas argon‐APPJ also promoted resolution but with a less pronounced trend (Figure 7D). These immunohistochemical observations suggest that plasma exposure may promote a more regulated healing trajectory characterised by earlier spatial normalisation of TGF‐β expression within the wound microenvironment.

## 4 Discussion

This study demonstrates that both nitrogen‐ and argon‐based APPJs improved wound closure, re‐epithelialisation and collagen deposition in diabetic rats. Although head‐to‐head comparisons were exploratory, the two exposures showed divergent profiles: nitrogen (RNS‐dominant) aligned angiogenesis with physiologic timing and accelerated TGF‐β normalisation, whereas argon (ROS‐dominant) promoted closure via oxidative signalling. These results highlight how gas chemistry directs different biological pathways in wound repair and nominate RNS‐dominant plasma as a promising parameter for refining dermatologic wound care.

### 4.1 Biological Mechanisms Underpinning Nitrogen APPJ Efficacy

Distinct healing profiles of nitrogen‐ versus argon‐based APPJs likely reflect differences in reactive species. Nitrogen APPJ generates abundant RNS, such as nitric oxide and nitrogen dioxide, which regulate endothelial activation, keratinocyte proliferation, fibroblast function and matrix remodelling—processes often impaired in diabetic wounds. Argon APPJ, in contrast, predominantly produces ROS that drive antimicrobial defence and redox signalling. These mechanisms are complementary rather than exclusive and the study was not powered for direct superiority testing but to delineate differential trajectories. Within this framework, RNS signalling appears particularly relevant for angiogenesis and matrix maturation, while ROS contributes antimicrobial and oxidative cues.

Our observations align with previous studies that have highlighted the therapeutic potential of CAP in wound management. For instance, Bolgeo et al. reported that CAP treatment reduced bacterial contamination and promoted wound healing in critically ill patients, emphasising its clinical applicability [30]. Similarly, Arndt et al. demonstrated that CAP induces the expression of key genes involved in wound healing, including IL‐6, IL‐8 and TGF‐β1, thereby enhancing tissue regeneration [31]. Together, these findings support the concept that distinct gas chemistries can drive complementary aspects of wound repair, emphasising the translational value of modulating RNS‐ versus ROS‐dominant exposures in plasma‐based dermatologic interventions.

### 4.2 Clinical Dermatology Perspectives

From a dermatological standpoint, the application of CAP has shown promise in treating various skin conditions. Gan et al. provided a systematic review of CAP applications in dermatology, noting its efficacy in wound healing and melanoma treatment [28]. Furthermore, Strohal et al. conducted a multicentre, randomised clinical trial comparing CAP‐jet therapy to best practice wound dressings, finding that CAP‐jet treatment resulted in faster wound healing and higher rates of complete healing in chronic wounds [32]. Our findings provide mechanistic context for clinical reports of plasma‐assisted wound healing by showing that reactive‐nitrogen–dominant exposure promoted angiogenesis aligned with physiologic timing and accelerated TGF‐β normalisation. This modulation is relevant for reducing fibrosis and scarring—major concerns in chronic wounds. Clinically, such trajectories imply earlier closure may reduce infection risk, fewer dressing changes, improved vascular normalisation and reduced likelihood of hypertrophic scarring. These wound‐level insights link preclinical biology with practical challenges in dermatologic management, underscoring gas chemistry as a modifiable parameter in APPJ therapy.

### 4.3 Future Directions and Clinical Implications

The distinct healing profiles of nitrogen‐ and argon‐based APPJs merit further translational investigation. Future studies should optimise parameters such as exposure duration, frequency and gas composition and assess combinations with growth factors, stem cells, or advanced dressings to enhance repair. Recent advances in biomaterial‐based wound therapies have also shown promising regenerative potential for diabetic wound repair. For example, hydrogel–exosome composite systems can modulate macrophage polarisation and promote angiogenesis and fibroblast migration, thereby accelerating tissue regeneration in diabetic wounds [33]. In addition, smart hydrogel dressings with real‐time wound monitoring capability and antibacterial activity have demonstrated the ability to enhance angiogenesis, collagen deposition and granulation tissue formation in diabetic wounds in preclinical studies [34, 35]. Decellularised extracellular matrix–based scaffolds have also emerged as a promising regenerative platform by providing bioactive microenvironments that support tissue remodelling and repair in regenerative medicine applications [36]. These advances highlight the growing interest in biomaterial‐based strategies for chronic wound management.

Gas chemistry thus emerges as a modifiable factor in wound care. Both RNS‐ and ROS‐dominant exposures improved diabetic healing through different biological pathways, supporting the need for clinical trials to confirm efficacy, safety and integration into standard care.

Plasma therapy has also been reported to exert antimicrobial effects in chronic wounds through the generation of reactive oxygen and nitrogen species [28]. Although the present study focused on host tissue responses—particularly angiogenesis dynamics and TGF‐β regulation—microbiological assessment of wound bacterial burden was not included in the experimental design. Because reduction of microbial load may contribute to improved wound healing outcomes, future studies should incorporate quantitative microbiological analyses to evaluate how nitrogen‐ and argon‐based plasma treatments influence wound bacterial burden and how antimicrobial effects interact with plasma‐mediated tissue repair processes.

### 4.4 Limitations

Several limitations should be acknowledged. First, all experiments were performed in a streptozotocin‐induced diabetic rat model, which may not fully capture the multifactorial pathophysiology of human chronic wounds. Second, mechanistic analyses were primarily limited to histology and immunohistochemistry. In particular, TGF‐β regulation was evaluated by immunohistochemistry without complementary quantitative molecular assays such as qPCR or Western blotting. Although this approach preserved spatial information within the wound microenvironment, it does not provide definitive transcriptional or protein‐level pathway validation. Future studies incorporating quantitative molecular analyses will be important to strengthen mechanistic interpretation. Third, only two working gases under fixed parameters were compared; broader gas admixtures and operating settings warrant further exploration. Fourth, the study was not powered for head‐to‐head superiority testing and between‐gas differences should be interpreted as mechanistic trends rather than definitive superiority. Fifth, sample sizes were further reduced by timepoint subdivision (*n* = 4 per group) and long‐term outcomes such as tensile strength or recurrence were not assessed. Future studies should incorporate prospective sample size estimation based on predefined primary endpoints and effect sizes derived from the present data to enable adequately powered validation of the exploratory trends observed here. Finally, only male rats were included to avoid potential estrous cycle‐related variability; future studies should incorporate both sexes to improve generalisability. Despite these limitations, the findings provide a preclinical foundation for further investigation of gas‐chemistry–modulated plasma therapy in diabetic wound healing.

Another technical consideration is that the nitrogen plasma generated higher discharge current and average power than the argon plasma under identical operating conditions. Although both plasmas remained within a non‐thermal and biocompatible temperature range, this difference in energy deposition may represent a contributing physical parameter that cannot be fully separated from gas chemistry under the current experimental design. Accordingly, the observed biological differences likely reflect a combined effect of gas chemistry and energy deposition, the relative contributions of which cannot be fully resolved in the current study. Because the present study aimed to compare gas‐dependent plasma outputs produced under identical device operating settings—reflecting a pragmatic device‐operating condition rather than a strictly controlled energy‐matched design—energy deposition was not independently normalised. Future investigations using power‐matched plasma conditions or controlled energy normalisation will be valuable for further disentangling the relative contributions of plasma chemistry and energy dose.

In addition, trace gas impurities may influence reactive species formation in atmospheric‐pressure plasma systems. Although ultra‐high‐purity gases (99.999%) were used in this study (O_2_ ≤ 2.0 ppm and H_2_O ≤ 0.91 ppm), small amounts of oxygen and water vapour can still participate in plasma–gas interactions and contribute to the formation of secondary reactive oxygen and nitrogen species such as OH radicals, NOx and hydrogen peroxide. Because both nitrogen and argon plasmas were generated using the same device configuration, gas supply and environmental conditions, impurity levels were consistent across groups. Nevertheless, ambient humidity and trace impurities may influence plasma chemistry in different experimental settings and future studies should systematically investigate how these parameters affect reactive species composition and therapeutic outcomes.

Furthermore, representative wound images for the healthy control group in Figure 3B are not available due to irreversible data corruption of the original image files, despite extensive recovery efforts. Although quantitative analyses and statistical comparisons involving the healthy control group were preserved and remain valid, the absence of representative images limits visual contextualisation of normal wound‐healing progression. Nevertheless, the quantitative trends remain consistent with established normal wound‐healing trajectories reported in the literature. Given that this group primarily serves as a reference for confirming successful induction of the diabetic chronic wound model, the main comparative findings remain largely interpretable, although the completeness of visual documentation is reduced. Future studies should adopt more robust data storage and verification strategies to minimise the risk of similar issues.

## 5 Conclusion

Both nitrogen‐ and argon‐based plasma treatments improved diabetic wound repair compared with untreated controls but resulted in distinct healing patterns. Nitrogen‐dominant exposure was associated with angiogenic dynamics that more closely aligned with physiological healing timing and with earlier normalisation of transforming growth factor beta expression, suggesting a trend towards a more regulated repair trajectory. In contrast, argon‐dominant exposure was associated with wound closure patterns consistent with oxidative pathway–mediated responses.

These results indicate that gas chemistry may represent a modifiable parameter influencing wound‐healing responses and suggest mechanistic differences between reactive nitrogen– and reactive oxygen–dominant plasma exposures. Cold plasma therefore represents a potential non‐pharmacologic adjunct strategy for difficult‐to‐heal diabetic wounds. Future clinical studies will be required to optimise treatment parameters and confirm safety and efficacy in patients.

## Funding

This work was supported by the Cathay General Hospital (CGH‐MR‐A113009, CGH‐MR‐A114017, CGH‐MR‐B113012) and the Ministry of Science and Technology, Taiwan (MOST‐107‐2221‐E‐072‐MY3).

## Ethics Statement

All animal experiments were conducted in accordance with the institutional guidelines for laboratory animal care and approved by the Institutional Animal Care and Use Committee (IACUC) of National Yang Ming Chiao Tung University (Approval No. NCTU‐IACUC‐106062). All efforts were made to minimise animal suffering and the number of animals used.

## Conflicts of Interest

The authors declare no conflicts of interest.

## Data Availability

The data that support the findings of this study are available from the corresponding author upon reasonable request.
